# Postoperative supraventricular tachycardia and polymorphic ventricular tachycardia due to a novel *SCN5A* variant: a case report of a rare comorbidity that is difficult to diagnose

**DOI:** 10.1186/s12872-020-01601-2

**Published:** 2020-07-02

**Authors:** Koichi Kato, Tomoya Ozawa, Seiko Ohno, Yoshihisa Nakagawa, Minoru Horie

**Affiliations:** 1 Department of Cardiovascular Medicine, Shiga University of Medical Science, Otsu, Japan; 2 Department of Bioscience and Genetics, National Cerebral and Cardiovascular Center, Suita, Japan; 3 Center for Epidemiologic Research in Asia, Shiga University of Medical Science, Otsu, 520-2192 Japan

**Keywords:** Case report, *SCN5A*, Ventricular fibrillation, Postoperative supraventricular tachycardia, Brugada syndrome, Cardiac conduction defect, Atrial septal defect

## Abstract

**Background:**

Loss-of-function mutations of human cardiac sodium channel gene *SCN5A* induce a wide range of arrhythmic disorders. Mutation carriers with co-existing conditions such as congenital heart diseases and histories of cardiac surgeries, could develop complex arrhythmic events that are difficult to diagnose.

**Case presentation:**

A 41-year-old Japanese male with a history of a surgical closure of an ASD presented impairment of consciousness by wide QRS tachycardia. Because the patient’s baseline ECG in sinus rhythm showed similar QRS axis with right bundle brunch block morphology, we suspected supraventricular tachycardia (SVT). During hospitalization, the patient developed polymorphic ventricular tachycardia that was induced by bradycardia. In an electrophysiological study, the SVT was identified as right atrial incisional tachycardia circulating around the scar in the right atrium.

The genetic analysis revealed a heterozygous *SCN5A* c.4037–4038 del TC, p. L1346HfsX38 variant. We diagnosed this patient as having progressive cardiac conduction disorder (PCCD) and polymorphic VT caused by the mutation. Incisional tachycardia with wide QRS morphology was a by-standing comorbidity related to the history of cardiac surgery which could miss lead the diagnosis.

The patient’s SVT was eliminated by radiofrequency catheter ablation. An implantable cardioverter defibrillator (ICD) was implanted for the secondary prevention of polymorphic VT. Cardiac pace-making therapy by the ICD to avoid bradycardia effectively suppressed the patient’s arrhythmic events.

**Conclusions:**

We treated a patient with a sodium channel gene variant. Co-existing SVT originated by a scar in the right atrium made the diagnosis extremely difficult. A multilateral diagnostic approach using an ECG analysis, an electrophysiological study, and genetic screening enabled effective combination therapy comprised of catheter ablation and an ICD.

## Background

Loss-of-function variants of human cardiac sodium channel gene *SCN5A* induce a wide range of arrhythmic disorders, such as Brugada syndrome [1, 2], dilated cardiomyopathy [3], and progressive cardiac conduction disorder (PCCD, also known as Lev-Lenègre syndrome) [4], which are known as sodium channelopathies.

Surgical closure for an atrial septal defect (ASD) is a well-established procedure with a good long-term outcome [5–8]. However, postoperative supraventricular tachycardia is a frequent sequela among ASD patients treated by surgical closure [6, 8].

Here we report the case of a patient with a multiple arrhythmia who carried an *SCN5A* frameshift mutation with a surgical repair history for ASD. We provide the details of the pathology of this complex case toward to goal of devising a better therapeutic approach.

## Case presentation

### Initial tachycardia attack in a case with a history of cardiac surgery

**Fig. 1:**
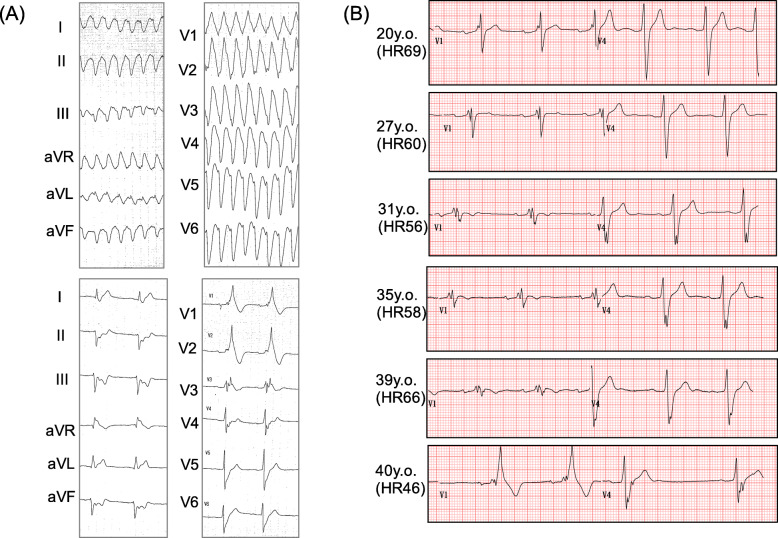
Initial tachycardia attack and the time-dependent progression of cardiac conduction defect in the patient, a 41-year-old male. **a** 12-lead ECGs of the first tachycardia event and the sinus rhythm. **b** ECGs recorded 20 years after the patient’s ASD closure

A 41-year-old Japanese male presented impairment of consciousness by wide QRS tachycardia (Fig. 1a). Although his systolic blood pressure has been kept around 60–70 mmHg during the tachycardia, his conscious level was moderately impaired to E1V2M4 in the Glasgow Coma Scale. At his arrival at a hospital, he was immediately sedated and intubated by emergency physicians who assessed his condition as a cardiogenic shock. The tachycardia self-terminated soon after the administration of the sedative. He had a history of a surgical closure of an ASD at 20 years old. After that surgery ~ 20 years earlier, his QRS interval had gradually increased to 198 ms, and in the 2 years prior to his presentation at our hospital, right bundle brunch block morphology became evident (Fig. 1b). Because the QRS axis of the tachycardia matched that of sinus rhythm, we suspected supraventricular tachycardia (SVT). After the initial assessment, we decided to observe his condition overnight in an intensive care unit under the sedated and intubated condition.

### In-hospital event

**Fig. 2:**
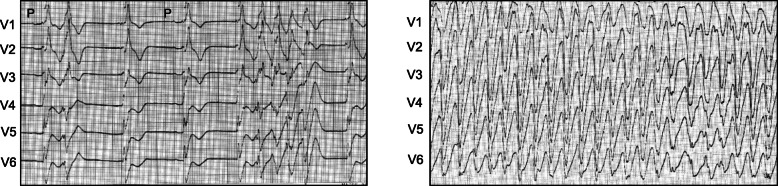
ECGs recorded during hospitalization. Sinus bradycardia and subsequent polymorphic ventricular tachycardia recorded under sedation (*left*). The polymorphic ventricular tachycardia which required a cardioversion (*right*)

In the intensive care unit, his sinus bradycardia seriously worsened during that night while he was under sedation, and he developed polymorphic ventricular tachycardia (VT) followed by ventricular fibrillation (Fig. 2) which required a cardioversion. As a marked sinus bradycardia continued, transcutaneous ventricular pacing was initiated, which increased his heart rate and successfully suppressed the recurrence of ventricular tachycardia. We considered that this arrhythmia was not identical with that observed in the initial event where the QRS morphology was stable and the blood pressure was kept above 60 mmHg. During these arrhythmic events, no prolonged QTc interval was recorded.

### Electrophysiological study for the patient’s SVT, and treatment

**Fig. 3:**
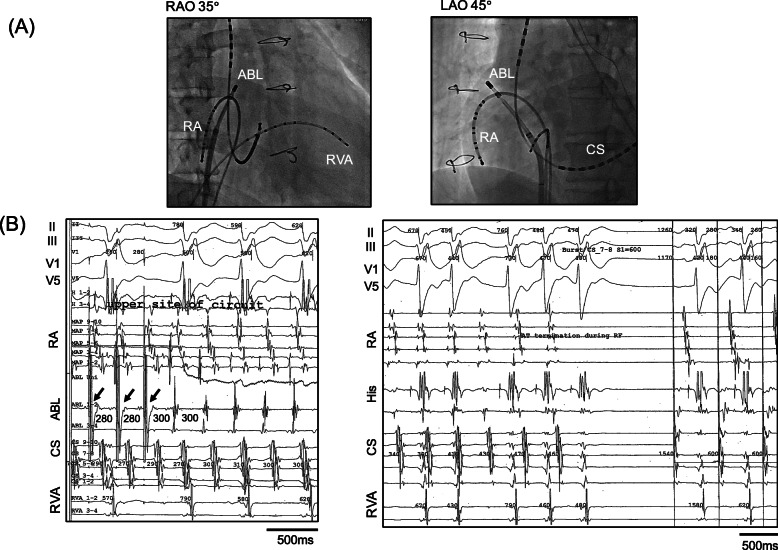
Electrophysiological study. **a** The right anterior oblique (RAO) and left anterior oblique (LAO) projections showing the electrode positions. The ABL electrode is at the upper edge of the scar in the right atrium. **b** The post-pacing interval from the ABL electrode at the upper edge of the scar matched the cycle length of the tachycardia (*left*). *Arrows* indicate the 280-ms burst pacing from the ABL electrode. Radiofrequency ablation promptly terminated the tachycardia (*right*)

In an electrophysiological study, programmed electrical stimulation (PES) from the right ventricle easily induced polymorphic ventricular tachycardia that sustained and degradated to ventricular fibrillation. Sustained monomorphic VT was never induced during the study. The patient’s SVT was identified as right atrial incisional tachycardia circulating around the scar in the right atrium (Fig. 3). The post-pacing interval at the upper end of the scar matched the tachycardia cycle length (Fig. 3a, b, left panel). At this site, radiofrequency catheter ablation successfully terminated the tachycardia (Fig. 3b, right).

To prevent sudden death by further attacks of ventricular fibrillation, the patient underwent implantable cardioverter-defibrillator (ICD) implantation. His bradycardia was treated by the pace-making therapy afforded by the ICD. Under an 80-bpm DDD-pacing condition, his heart beat was fully paced, and the polymorphic ventricular tachycardia was successfully suppressed. During 18 months’ follow-up, he has never experienced further arrhythmic events and there were no adverse events related to interventions he received. He returned to work as before, and is satisfied with the treatment results he received.

### Genetic analysis

**Fig. 4:**
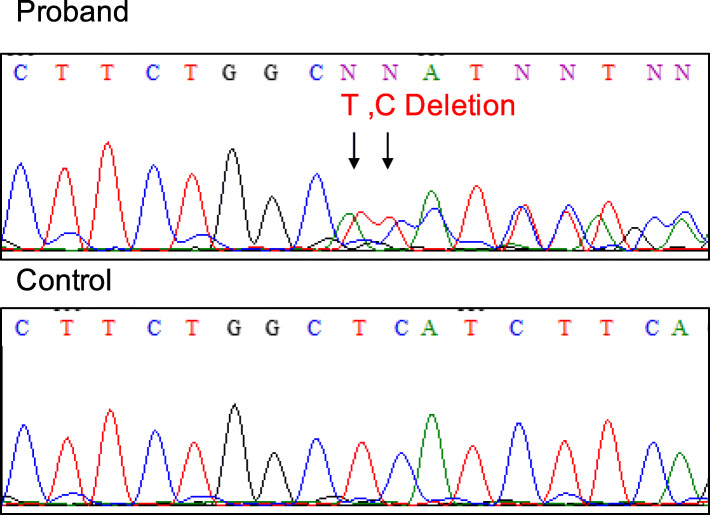
The genetic analysis results. Genomic DNA sequence of SCN5A from the patient (*top*) and a control sample (*bottom*)

The patient’s severe sinus bradycardia, progressive QRS widening, high voltage amplitude of R’ wave in the right precordial leads, and recurrent ventricular arrhythmias indicated that the patient had cardiac sodium channelopathy like PCCD and Brugada syndrome. We then performed genetic screening for inherited arrhythmias by targeted gene sequencing. The genetic analysis revealed a heterozygous *SCN5A* c.4037–4038 del TC, p. L1346HfsX38 variant (Fig. 4). The 2-bp deletion in exon 23 of *SCN5A* induced the truncation of mRNA, leading to nonsense-mediated mRNA decay (NMD), and thereby decreasing the expression level of the cardiac sodium channel Nav1.5. The same variant was also detected in the genomic DNA from the patient’s asymptomatic mother. Her ECG manifested only mild QRS widening with 130 ms QRS interval and slight sinus bradycardia (HR 50-56 bpm). This variant had not been reported in large genomic databases such as gnomAD (https://gnomad.broadinstitute.org) and TOGOVAR (https://togovar.biosciencedbc.jp/).

In the American College of Medical Genetics and Genomics (ACMG) guideline, this variant is classified as “likely pathogenic” with one PVS1 (null variant) and one PM2 (not observed in population databases).

We diagnosed this patient as having a PCCD and polymorphic ventricular tachycardia due to the *SCN5A* loss-of-function variant. Postoperative SVT with wide QRS morphology was a by-standing comorbidity related to the history of cardiac surgery which could miss lead the diagnosis.

## Discussion and conclusions

We presented a patient with a heterozygous *SCN5A* frameshift variant and a history of a surgical ASD closure. The identified variant, p. L1346HfsX38, was not reported previously. Because the frameshift variant was in exon 23 of *SCN5A*, which normally consists of 28 exons, we suspected that the variant caused a truncation of mRNA, resulting in the decreased expression level of Nav1.5 due to NMD [9]. The patient’s sinus bradycardia, cardiac conduction disturbance with severe QRS widening, and ventricular fibrillation were all compatible as phenotypes of Brugada syndrome [10]. However, type 1 Brugada ECG findings were not evident because of the patient’s complete right bundle brunch block. The fact that the patient’s mother also carried the same variant but remained asymptomatic was not surprising, because 90% of Brugada syndrome patients are male [10, 11].

In the last decade, the concept that sodium channelopathies are not pure arrhythmogenic disorders but rather are cardiomyopathies with a strong arrhythmogenesis has been widely accepted. An association between these variants and congenital structural heart diseases has not been demonstrated. In our patient’s case, we could not conclude that the patient’s ASD was caused by his *SCN5A* variant.

Trans-catheter ASD closure with a device has gained acceptance, but surgical closure remains the first-line strategy with excellent long-term outcomes [8]. Sinus node dysfunction requiring a pacemaker implantation was reported in approx. 4% of patients after a surgical ASD closure in a long-term follow-up study [12], but severe QRS widening, bundle brunch blocks, and life-threatening ventricular arrhythmias are extremely rare. In our patient’s case, the sinus bradycardia had worsened during 20 years after his ASD surgery, and complete right bundle brunch block with severe QRS widening was observed as well. This drastic change was not plausible as a late complication of surgical repair for ASD.

Postoperative SVT is commonly seen after a cardiac surgery; not only for ASD repair, but for all types of congenital heart disease [13]. Our patient’s arrhythmia was observed to be circulating around the incision in the right atrium (which is known as incisional tachycardia), and radiofrequency ablation at the top of the incision successfully eliminated the arrhythmia. Due to the impaired cardiac conduction by an *SCN5A* variant, this often-observed arrhythmia presented a rare clinical manifestation that was initially difficult to interpret.

The sedation in the intensive care unit possibly triggered his polymorphic VT by worsening bradycardia. However, considering the rapid progression of CCD in the last 2 years, we are convinced that he would have the similar adverse event in very near future even without sedation. When it comes to the choice of implantable cardiac device, although this patient’s polymorphic VT was successfully suppressed by controlling bradycardia, we chose ICD rather than simple DDD pacemaker. It is because of the high inducibility of polymorphic VT by PES from the right ventricle. PES-induced ventricular tachycardia is one of the predictors of lethal events of Brugada syndrome [14] even though there is still a controversy [15]. We concluded that ICD implantation was not an overtreatment for this patient’s case.

Currently, 97% of the patient’s heart beats are stimulated by dual chamber pacing by the ICD. At the time of implantation, we did not expect that a setting preferring patient’s intrinsic ventricular beats had a significant advantage over right ventricular stimulation for cardiac synchronicity because his own QRS was extremely widened. However, considering his age, it is also necessary to optimize the pacemaker setting by assessing hemodynamics with or without right ventricular pacing, as well as preventing bradycardia.

As a differential diagnosis, arrhythmogenic right ventricular cardiomyopathy and bundle branch reentry VT can be stated. But cardiac magnetic resonance imaging could not detect late gadolinium-enhancement in either left or right ventricular cardiac muscle. Sustained monomorphic VT, which was suggesting bundle brunch VT, could not be induced by repetitive PES. Furthermore, we detected no genetic variant in desmosomal genes, typical ARVC causative genes.

In conclusion, multiple coexisting arrhythmogenicities induced repetitive arrhythmias difficult to diagnose in our patient with a sodium channelopathy. A precise understanding of this patient’s pathology by a multilateral approach using an ECG analysis, an electrophysiological study, and genetic screening enabled effective combination therapy comprised of catheter ablation and an ICD.

## Data Availability

Most of data analyzed during this study are included in this published article. Full EP study records are not publicly available but are available from the corresponding author on reasonable request.

## Abbreviations

ASD: Atrial septal defect
PCCD: Progressive cardiac conduction defect
SVT: Supraventricular tachycardia
VT: Ventricular tachycardia
NMD: Nonsense mediated mRNA decay
PES: Programmed electrical stimulation
